# On the Role of Physical Interaction on Performance of Object Manipulation by Dyads

**DOI:** 10.3389/fnhum.2017.00533

**Published:** 2017-11-07

**Authors:** Keivan Mojtahedi, Qiushi Fu, Marco Santello

**Affiliations:** School of Biological and Health Systems Engineering, Arizona State University, Tempe, AZ, United States

**Keywords:** social interaction, interpersonal action, coordination, physical interaction, object manipulation

## Abstract

Human physical interactions can be intrapersonal, e.g., manipulating an object bimanually, or interpersonal, e.g., transporting an object with another person. In both cases, one or two agents are required to coordinate their limbs to attain the task goal. We investigated the physical coordination of two hands during an object-balancing task performed either bimanually by one agent or jointly by two agents. The task consisted of a series of static (holding) and dynamic (moving) phases, initiated by auditory cues. We found that task performance of dyads was not affected by different pairings of dominant and non-dominant hands. However, the spatial configuration of the two agents (side-by-side vs. face-to-face) appears to play an important role, such that dyads performed better side-by-side than face-to-face. Furthermore, we demonstrated that only individuals with worse solo performance can benefit from interpersonal coordination through physical couplings, whereas the better individuals do not. The present work extends ongoing investigations on human-human physical interactions by providing new insights about factors that influence dyadic performance. Our findings could potentially impact several areas, including robotic-assisted therapies, sensorimotor learning and human performance augmentation.

## Introduction

An important component of social behavior is the ability to coordinate actions with another person without verbal communication. Such coordination has been investigated extensively using tasks that impose visual or auditory coupling between two agents, such as finger tapping and pendulum swing, to characterize social coordination and underlying neural mechanisms (Schmidt et al., 1998; Sebanz et al., 2006; Richardson et al., 2008; Konvalinka et al., 2010; Fine and Amazeen, 2011; Yun et al., 2012; Masumoto and Inui, 2013, 2015; Solnik et al., 2015, 2016). This type of tasks has no physical contact or physical interaction between the two coordinating agents. However, physical interaction is one of the most important and common features of human motor behaviors, such as handing over objects, hand-shaking, dancing with a partner, moving heavy objects, or assisting movement of a patient undergoing physical rehabilitation. Despite the prevalence of physical interactions in our daily lives, the effect of physical coupling on motor coordination between two agents remains largely unknown.

Only a few studies have examined the difference in performance when executing the same task by comparing single-agent with physically connected dual-agent configurations. Reed and Peshkin (2008) demonstrated that, when two subjects are asked to rotate a crank together to reach a target, they perform the task faster than when acting alone. Similarly, when two subjects were asked to track the same moving target while holding linked robot handles, performance was better than when each subject performed the task alone (Ganesh et al., 2014). However, a potential confound of these studies is that the subjects in single-agent configuration performed the task unimanually, whereas the dual-agent configuration consists of two hand/arms that both physically contributed to the task. Therefore, the improvement in performance (e.g., speed or accuracy) associated with dual-agent configuration may be, at least partially, due to the addition of an end-effector, instead of the existence of physical coupling or interpersonal coordination. Indeed, van der Wel et al. (2011) compared motor performance of dual-agent with bimanual single-agent configurations using a pole swing task and showed that dyads performed at the same level as individuals. However, this study assumed no inter-personal difference between two paired agents when performing the task individually, and quantified individual performance of only one of the paired agents.

Another study (Eils et al., 2017) investigated a whole-body joint balance task as dyads (leader and follower) stood on a board/surface and had to guide a virtual ball through a maze and towards a virtual hole as fast as they could by jointly shifting their weight on the board. This study consisted of three visual conditions whereby visual access of follower to both leader and maze was manipulated. The completion time of the maze task was measured across these three conditions: (1) no visual access to the leader nor to the maze; (2) visual access to the leader but not the maze and (3) full visual access to both the leader and the maze. The completion time correlated with the amount of visual feedback such that it was longest when follower relied only on haptic information (no visual access to the leader nor to the maze). Conversely, performance was better when visual access to the leader was provided, with the best performance being when the follower had full visual access to both leader and maze. Other studies (Knoblich et al., 2011; Candidi et al., 2015; Pezzulo et al., 2017) of physical interactions have also shown that online sensorimotor communication and adaptation help individuals in aligning their task representations and improving joint action performance. This “co-representation” entails the sharing of internal representations of both the task and mental state of others (Sebanz et al., 2006; Knoblich et al., 2011; Buneo et al., 2014). It is worth mentioning that, to date, the mental and neural representation in physical interaction has not been systematically explored. However, there are non-physical interaction studies that have investigated the interdependencies of neural processes with regard to the performances and adaptations via hyperscanning technique—recording brain activity simultaneously from two people—while two participants interact with each other. These studies have provided insight into both intrapersonal and interpersonal neural processes through dual EEG (Dumas et al., 2011; Babiloni and Astolfi, 2012), dual fMRI (Saito et al., 2010) and dual fNIRS (Jiang et al., 2012).

Another overlooked factor in studies that involve physical interaction is handedness. There is extensive evidence that dominant and non-dominant hands are specialized in different aspects of motor control (Sainburg, 2014). However, most previous work has examined only one handedness configuration in dual-agent conditions, i.e., which hand was used by each agent in the joint actions. For instance, van der Wel et al. (2011) examined pairing of dominant and non-dominant hands, whereas other studies focused only on pairing of two dominant hands (Reed and Peshkin, 2008; Ganesh et al., 2014). Therefore, the extent to which handedness may play a role in performance by physically-coupled dyads remains unknown.

To address these gaps and improve our understanding of the role of joint actions with physical couplings on motor performance, we designed a novel object manipulation task that required the coordination of two end-effectors, i.e., hands from one or two agents. Object manipulation is commonly used to study unimanual sensorimotor control (Johansson and Flanagan, 2009). Extensive evidence suggests that unimanual object lifting is mediated by predictive control based on internal models of the object properties (Flanagan and Wing, 1997; Salimi et al., 2000), whereas unimanual object holding (i.e., to maintain balance) may rely on reactive control using on-line sensory feedback to control multi-digit forces (Johansson and Birznieks, 2004; Johansson and Flanagan, 2009). In the current study, two end-effectors are physically required to lift and hold the object horizontally. Nevertheless, the above sensorimotor control framework could also be applied to the bimanual configuration (Swinnen and Wenderoth, 2004; Fairhurst et al., 2014) since the central nervous system (CNS) has full knowledge of the object and involved end-effectors, similar to unimanual scenarios. In contrast, sensorimotor processes underlying object manipulation by two agents could be considered as more challenging or complex, as the sensorimotor system of each agent may not be able to accurately predict the sensory consequences arising from joint motor action. Therefore, we quantified the inter-personal coordination during physically-coupled joint actions by comparing task performance in dyadic configuration with individual performances in bimanual (i.e., intrapersonal) configuration. Furthermore, our experimental conditions were designed to include different combinations of handedness in dyadic configurations. It has been proposed that dominant and non-dominant limbs are specialized in predictive and impedance control, respectively, due to hemispheric lateralization (for review see Serrien et al., 2006; Sainburg, 2014).

Although both predictive and impedance control are likely to be involved in the control of each limb, the extent to which these two control mechanisms contribute to the final motor output appears to be asymmetrical (Sainburg, 2014). Therefore, when right-handed subjects are tested, it is expected that the dominant (i.e., right) limb has advantages in predictive control, whereas the non-dominant (i.e., left) limb has advantages in impedance control. Indeed, in unimanual rapid reaching tasks, the dominant arm is superior in stabilizing movement trajectory, whereas the non-dominant arm is better at reducing error at the final position (Sainburg and Kalakanis, 2000; Bagesteiro and Sainburg, 2002; Duff and Sainburg, 2002; Wang and Sainburg, 2007; Shabbott and Sainburg, 2008; Tomlinson and Sainburg, 2012; Mutha et al., 2013). When reaching with a robotic manipulandum, the dominant arm has been shown to perform better in a predictable novel force field, whereas the non-dominant arm performs better in an unpredictable force field (Yadav and Sainburg, 2014). In object manipulation, the notion of lateralization of control mechanisms is supported by analyzing grip forces in object lifting tasks, which demonstrated that the non-dominant hand relies more on the feedback-driven force corrections than the dominant hand (Rezvanian et al., 2014). Lastly, the effect of handedness emerges also during bimanual tasks. When two hands are used together, the dominant hand takes on the manipulative role while the non-dominant hand is used for posture stabilization, e.g., unscrewing the lid of a jar (Guiard et al., 1983; Swinnen et al., 1991; Wiesendanger and Serrien, 2001; Swinnen and Wenderoth, 2004). Based on above considerations, we expected different hand pairings may also influence dyadic performance in this study, and that this influence would be sensitive to the task phase, i.e., static vs. dynamic.

We tested the following hypotheses: (1) performance of the manipulation task by dyads and single agents (bimanual manipulation) would be comparable; (2a) dyadic performance in paired dominant hand configuration would be better than paired non-dominant hand configuration when moving an object; and (2b) dyadic performance in paired non-dominant hand configuration would be better than paired dominant hand configuration when holding an object.

## Materials and Methods

### Subjects

Seventy-two right-handed subjects (age: 19–31 years, 43 males) participated in the experiment. We assessed hand dominance using the Edinburgh Handedness Inventory (Oldfield, 1971). Subjects had no history or record of neurological disorders and were naïve to the purpose of the studies. Subjects gave informed written consent to participate in the experiments, which were approved by the Institutional Review Board at Arizona State University and were in accordance with the Declaration of Helsinki.

### Experimental Apparatus

**Figure 1:**
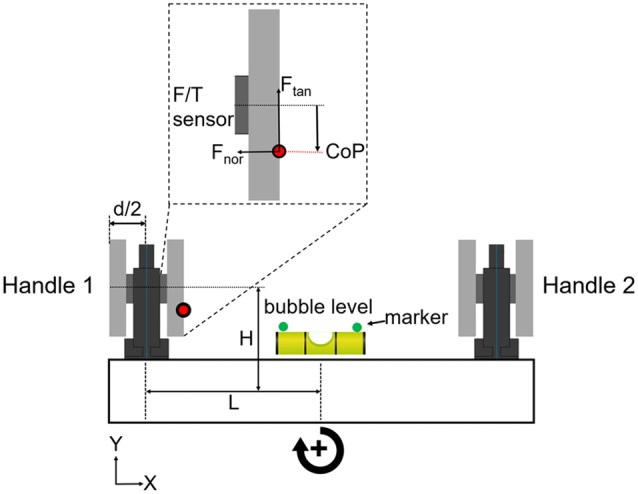
Grip device and experimental protocol. The grip device consisted of two identical handles mounted on horizontal base. Subjects could choose digit placement on two long graspable surfaces. Force/torque (F/T) sensors were mounted under the graspable surfaces to measure the x-, y- and z-components of forces and torques of the thumb and other fingers (Fu et al., 2010; Mojtahedi et al., 2015). Thumb and fingers grasped the inner and outer sides of each handle. The tilt (error) of the device was shown to the subjects by the bubble level placed in the mid-point of the horizontal base. Object height and error were measured by a motion tracking system using infrared markers (green circles) on each side of the bubble level. The parameters “L” plus “d/2” denote the horizontal moment arm, and “H” denotes the vertical moment arm. These parameters were used to formulate the mechanical model of the U-shaped grip device (see Supplementary Material). We used the output of each F/T sensor to measure digit(s) center of pressure (*CoP*; red dot), tangential and normal forces (*F*_tan_ and *F*_nor_) on each side of the handle (inset shows these variables measured on the thumb side of handle 1). Clockwise and counter clockwise object rotations are defined as positive and negative directions, respectively, and the same convention is used for the performance error (object tilt relative to horizontal).

We asked subjects to grasp a rigid U-shape object with all digits. The object consisted of two grip devices mounted on a horizontal base (Figure 1). The object was designed to have a symmetrical mass distribution with the center of mass located at the mid-point of the horizontal base. The object’s weight was 1088 g. The object’s height, length, and width were 185, 390 and 45 mm, respectively. A bubble level was placed in the middle of device. Two infrared markers (green circles, Figure 1) were glued on the sides of the bubble to record the height and tilt of device. Object kinematics was recorded using a motion tracking system (Phase Space; sampling frequency: 480 Hz). Forces and torques exerted by the thumb and all fingers on each handle were measured by two 6-axis force/torques sensors (ATI Nano-25 SI-125–3; sampling frequency: 1 kHz). As this article focuses on manipulation performance, and for the sake of brevity, force and torque data analyses will be presented as part of a follow-up study.

### Experimental Protocol

**Figure 2:**
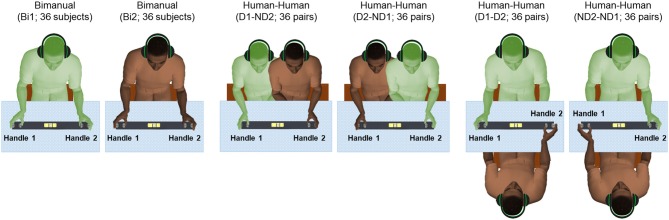
Experimental protocol. There are six conditions: bimanual (Bi1 and Bi2), dominant hand and non-dominant hand (D1-ND2 and D2-ND1), both dominant hand (D1-D2), and both non-dominant hand (ND2-ND1). Subjects in all conditions received auditory cues to initiate movement of the grip device. We defined “Handle 1” as the handle used by the participant’s dominant hand in all conditions with the exception of the condition where both subjects used their non-dominant hand (ND2-ND1) as “Handle 1” used by ND2.

**Figure 3:**
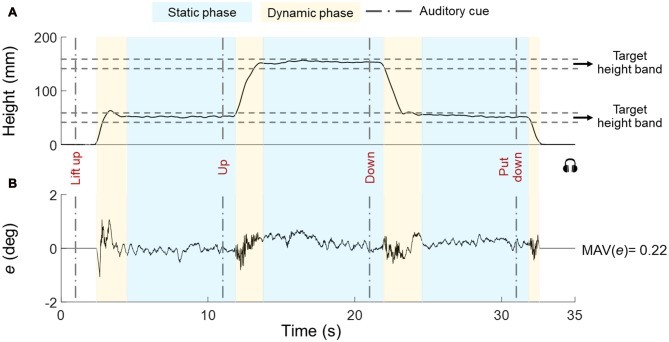
Representative trial for height and error (tilt). The object vertical position and error (e) are shown in **(A,B)**, respectively. The timeline of auditory cues is denoted by vertical dashed lines. The boundaries of the target height subjects had to position the object at (height band) are shown by horizontal dashed lines. Dynamic and static phases are denoted by yellow and blue boxes, respectively. Data are from one individual in Bi group.

Each subject was asked to use a whole-hand grasp and vertically lift the object using either both hands (one on each handle, *Bimanual*; Figure 2), or perform the same task by cooperating with another subject by grasping one handle with the right or left hand (*Human-Human*; Figure 2). Auditory cues delivered through headphones signaled the onset and offset of dynamic and static phases, i.e., moving the object upward or downward, and holding the object still, respectively (Figure 3). We used two parallel rectangular bands as visual cues denoting the minimum and maximum height (target height bands) within which the object had to be positioned and held.

We asked subjects to reach and keep their hand(s) close to handle(s) before the beginning of each trial. Subjects waited for the first auditory cue (*“lift up”*), after which they closed their hand(s) on the object and lifted the object. We instructed subjects to grasp the object with the thumb and all fingertips on the graspable surfaces, lift the object at a natural speed while keeping it horizontal until they reached the first target height band (45–55 mm), hold it there until hearing the next auditory cue (*“up”*), lift the object to the second target height band (145–155 mm) and hold it there until hearing the next auditory cue (*“down”*), bring down the object to the first target height band and hold it there. Until hearing the last auditory cue (*“put down”*) that signaled the replacement of the object on the table (Figure 3). The interval between auditory cues was 10 s. The experimental task goal was to keep the U-shaped object as horizontal as possible across all task phases while staying within the height bands. We instructed subjects to visually monitor the bubble level as feedback for controlling the orientation of the object throughout the task. Each trial lasted 31–33 s and subjects were given 30–60 s rest between trials to prevent fatigue.

Participants (*n* = 72) were randomly selected to create 36 subject pairs (dyads). All the subjects were randomly paired based on their available times. None of the participants of each dyad had met before. The gender distributions across dyads consisted of 16, 9 and 11 pairs for male-male, female-female and female-male pairs, respectively. For each pair of subjects, there were a total of six experimental conditions (Figure 2). Each participant performed a bimanual condition (Bi1 and Bi2; respectively) to measure baseline manipulation performance. Additionally, we tested two experimental conditions where the two partners sat side by side, with one partner using his/her dominant (right) hand and the other using his/her non-dominant (left) hand (D1-ND2 and D2-ND1, respectively). Lastly, we tested two conditions in which partners sat in front of each other so that both participants could use their dominant or non-dominant hand (D1-D2 and ND2-ND1, respectively). Note that in all dyadic conditions, the thumb of either right or left hand was located inside the U-shape device to match the hand configuration tested in the bimanual condition. This was an important consideration of our design as we used the bimanual condition (where both thumbs are located inside the U-shape device) as baseline for comparing performance with all dyadic conditions (for details see “Statistical Analysis” section).

**Figure 4:**
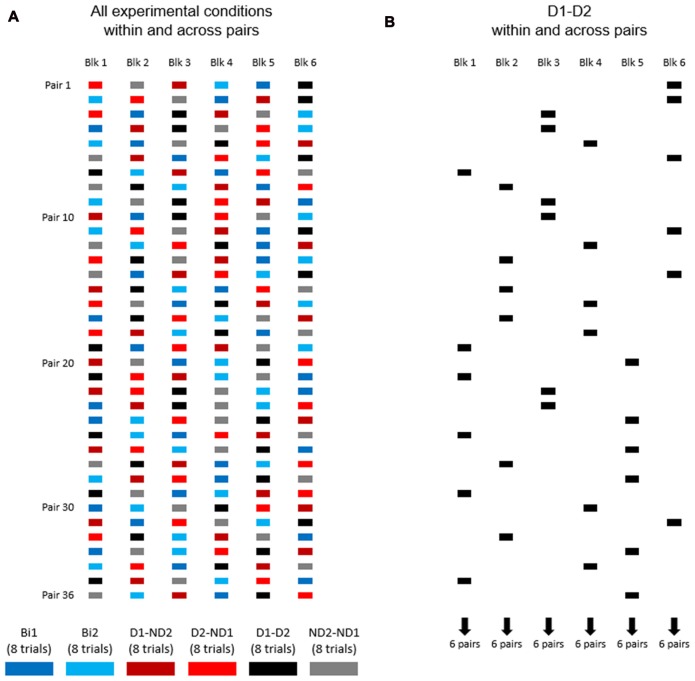
The order of experimental conditions within and across subject pairs. **(A)** Experimental conditions are color coded. Each pair of participants performed one block of eight consecutive trials per experimental condition, for a total of 48 trials (6 blocks × 8 trials). The order of presentation of experimental conditions was counterbalanced across pairs of participants. **(B)** The order of D1-D2 experimental condition within and across subject pairs.

Figure 4A shows the distribution of experimental conditions within and across subject pairs. Each pair of participants performed one block of eight consecutive trials per experimental condition, for a total of 48 trials (6 blocks × 8 trials). The order of presentation of experimental conditions was counterbalanced across pairs of participants (Figure 4A). During data collection of the bimanual condition (one participant), we asked the other participant to leave the room and wait outside.

All subjects were instructed to minimize object tilt throughout all task phases. Thus, for the Bi condition, subjects were asked to coordinate their hand movements and torques as accurately as possible. Subjects in the dyad conditions were asked to cooperate with each other to control object orientation. All subjects were reminded of the task goal before starting the first trial in each condition.

### Data Processing and Experimental Variables

Figure 3 shows data from a representative trial (H-H group) and performance variables.

1. *Task phases*. We defined a dynamic and static phase for each target height (Figure 3A). The onset of the dynamic phase was defined as the first time point at which the vertical position of the object center changed ±5% relative to the previous vertical position averaged across 800 ms and stayed above that threshold for 600 ms. Similarly, the onset of the static phase was defined as the first time point after which the object vertical position computed over the past 600 ms remained within ±5% relative to the vertical position averaged across the following 800 ms.
2. *Performance error*. For each trial, we quantified performance error (*e*) as the mean absolute value (MAV) of object tilt relative to the horizontal (MAV(*e*)) to capture the average quality of performance across all static and dynamic phases of each trial (Figure 3B).

### Statistical Analysis

All the statistical analyses were performed in the software of Statistical Package for the Social Sciences (SPSS). The results of each statistical analysis described below are reported in specific sections in the Results. We analyzed dynamic and static performances separately.

#### Learning Effect in Performance within Block for all Conditions

**Figure 5:**
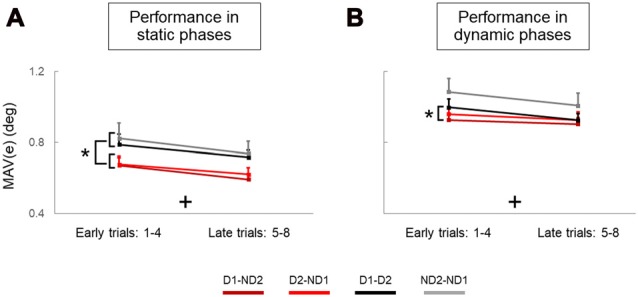
Performance error of static and dynamic phases across trials and experimental conditions. **(A,B)** are mean absolute values of error (MAV(*e*)) measured on early and late trials in static and dynamic phases, respectively. Data are means averaged across all subjects. Vertical bars denote standard errors of the mean. The symbol “+” indicates a statistically significant *Trial* effect. The asterisk denotes statistically significant differences (*p* < 0.05).

To assess learning within each block of trials (i.e., experimental condition; Figures 5A,B), we divided the eight trials into “Early trials” (trials 1–4” and “Late trials” (trials 5–8). We performed analysis of variance (ANOVA) with repeated measures on MAV(*e*) using one between-subject factor, *Group* (2 levels: Bi1 and Bi2), and two within-subject factors: *Trial* (2 levels: trials 1–4 and trials 5–8) and *Condition* (5 levels: Bi, D1-ND2, D2-ND1, D1-D2, ND2-ND1).

#### Performance within Block Across Dyadic Conditions

All dyadic conditions were designed to quantify the effect of handedness and configuration of each participant (Figure 2). We note here that the choice of our dyadic conditions was constrained by the criterion of having the thumb of the hand grasping the handle inside the U-shaped device to allow comparison with the bimanual condition. Therefore, we did not test “D1-D2” and ND2-ND1 in the “side by side configuration” or “D1-ND2” and D2-ND1 in the “face-to-face configuration” conditions as these would not have been comparable with neither the bimanual condition nor other dyadic interactions. However, elimination of these four configuration conditions create a confounding factor. Specifically, moving from side-by-side to face-to-face configurations affects the dominance factor, as there is no equivalent of neither the D-D nor ND-ND of the face-to-face configuration that can meet the above-mentioned grasp type criterion in the side-by-side configuration—and vice versa for the D1-ND2 and D2-ND1. To address this confounding factor, we performed these two analyses:

1. Repeated measure ANOVA analysis: we performed repeated measures ANOVA using two within-subject factors: *Trial* (2 levels: trials 1–4 and trials 5–8), and *Handedness* (2 levels: D1-D2 and ND2-ND1). Note that these two dyadic conditions consist of face-to-face configurations and there was no confounding factor in this analysis.
2. Linear mixed model analysis: we had the nested or ill-posed problem between the factors of configuration and handedness when we tested the existence of configuration effect no matter how we approached it due to the confounding factor.

To account for the hierarchical structure in our design—subjects nested within dyads (Kenny et al., 2006)—we used a repeated-measures analysis in a mixed-effects model framework to analyze the effect of categorical effects of *Configuration*, *Handedness*, *Trial* and interaction on dyadic behavioral performance. To this end, we included random intercepts for the levels of individual subjects and dyads, as well as accounting for dyad membership of each subject. Mixed model covariance structures were specified as first-order autoregressive. This choice of structure was employed based on the assumption that any correlation in residuals between levels of our factors was identical across factor levels. We specified *Configuration* (4 levels: D1-ND2, D2-ND1, D1-D2, ND2-ND1), *Handedness* (2 levels: dominant and non-dominant) and *Trial* (2 levels: trials 1–4 and trials 5–8) as Repeated variables. We chose dyadic performance as the dependent variable. Fixed effects were *Configuration*, *Handedness* and *Trial*. Random intercepts were specified for each dyad. This approach allows us to account for the fact that statistical model residuals in our design occur within dyad, which emerges from individual subject membership to a group. We used Maximum Likelihood (ML) for mixed model estimation and Bonferroni for pairwise comparisons. Normality of mixed-effect model residuals were assessed using scatter and quantile-quantile plots of the models’ residuals as compared to the fitted values. When designing and testing each mixed model, we always started with the model containing both Configuration and Handedness factors, and their interaction. The model was subsequently reduced until only significant terms were remaining (West et al., 2015).

#### Performance Across Blocks for Each Experimental Condition (Practice Effect)

**Figure 6:**
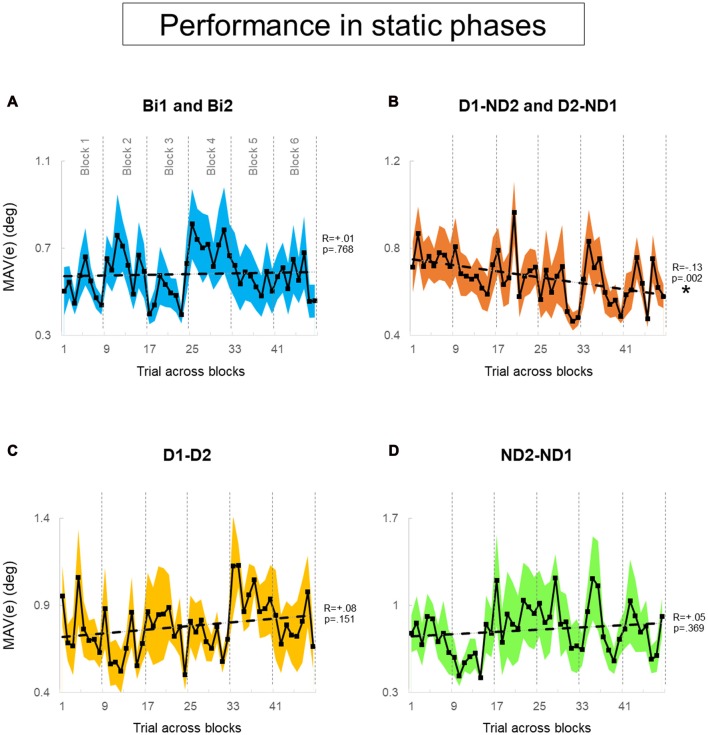
Performance error of static phase. **(A–D)** MAV(*e*) across trials and blocks for bimanual conditions, dominant and non-dominant hand conditions, both dominant hand condition and both non-dominant hand condition, respectively. There is six blocks and each block has eight trials. We tested 12 subjects and 12 pairs for each block in **(A,B)**, respectively. We tested six dyads for each block in **(C,D)**. Data are means averaged across all subjects or pairs. The shaded area around the main plot denote standard errors of the mean. The dash line plotted is the best fitted regression to test whether there is a significant trend for MAV(e) across trials and blocks. The asterisk denotes statistically significant relationship (correlation) between performance and trial (*p* < 0.05).

**Figure 7:**
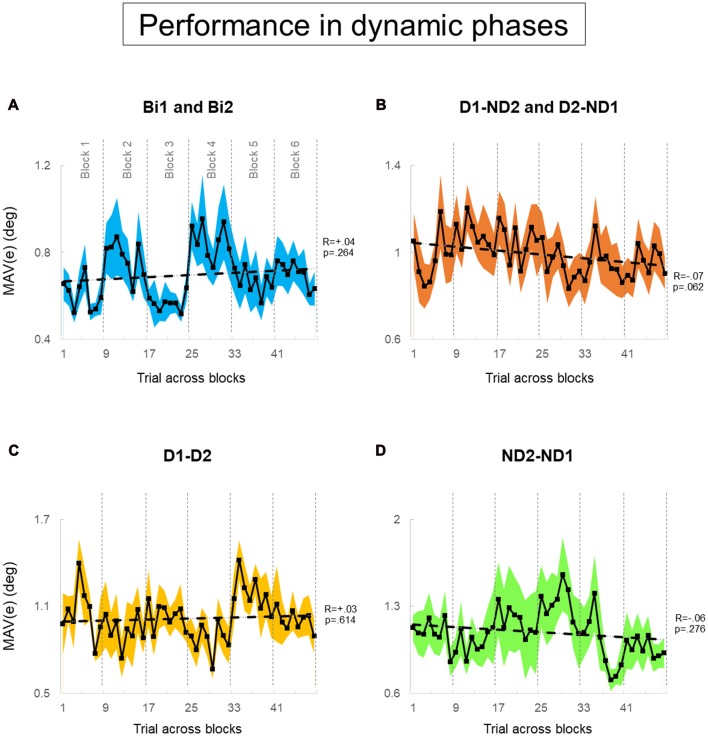
Performance error of dynamic phase. **(A–D)** MAV(*e*) across trials and blocks for bimanual conditions, dominant and non-dominant hand conditions, both dominant hand condition, and both non-dominant hand condition; respectively. There are six blocks, and each block consists of eight trials. We tested 12 subjects and 12 pairs for each block in **(A,B)**, respectively. We tested six dyads for each block in **(C,D)**. Data are means averaged across all subjects or pairs. The shaded area around the main plot denote standard errors of the mean. The dash line plotted is the best fitted regression to test whether there is a significant trend for MAV(e) across trials and blocks.

Our experimental design (Figure 4A) was also motivated by the goal of assessing learning that might have occurred as a function of amount of practice of the manipulation task. As an example, Figure 4B shows condition D1-D2 being presented at different points in the presentation sequence of experimental conditions across subject pairs, e.g., six pairs of subjects were tested on block 1, six different pairs on block 2 and so forth up to block 6^1^. Therefore, this design allowed us to quantify whether subjects tested on a given experimental condition later in the experiment might have performed differently than those exposed to the same condition at earlier points, i.e., whether participants might have benefited from having practiced the manipulation task in other experimental conditions. To assess learning effect across blocks for each condition, we performed linear regression analysis on MAV(*e*) across 48 trials (6 blocks × 8 trials; Figures 6A–D, 7A–D). These analyses were performed separately for the task dynamic and static phases.

#### Effect of Gender on Performance

First, we performed one-way ANOVA on MAV(e) of bimanual condition with one between subject factor, *Gender* (2 levels: male, female). Second, we performed ANOVA with repeated measures on MAV(e) only for dyadic conditions using one between-subject factor, *Gender-combination* (3 levels: male-male, female-female, and male-female) and two within-subject factors, *Trial* (2 levels: trials 1–4 and trials 5–8) and *Condition* (4 levels: D1-ND2, D2-ND1, D1-D2, ND2-ND1).

#### Influence of Physical Interaction on Performance of Individual Agents

**Figure 8:**
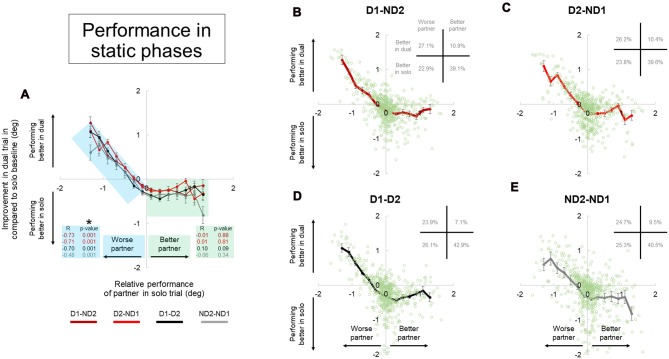
Performance during dual interaction vs. during bimanual interaction in static phase. Influence of all dual interaction with respect to bimanual interaction is shown in **(A)**. The improvement in task performance in each subject for each dual trial was plotted against the relative performance of their partner. The dual trial improvement was measured by the change in tilt error by a subject during a single trial compared to his individual tilt error in the correspondent dual trial. The positive and negative abscissas in *x*-axis corresponds to a better (superior) performing partner and a worse (inferior) performing partner; respectively. The positive and negative abscissas in *y-axis* corresponds to an agent who is performing better in dual trial and performing better in solo trial; respectively. The green and blue shaded boxes are for better and worse partner; respectively. Average performances and all the data points are shown for each dual condition in **(B–E)**. Furthermore, the frequency of occurrence of data points in each quadrant is also shown in top right of each plot in **(B–E)**. Data are means averaged across all subjects. Vertical bars denote standard errors of the mean. The asterisk denotes statistically significant correlation or relationship (*p* < 0.05).

**Figure 9:**
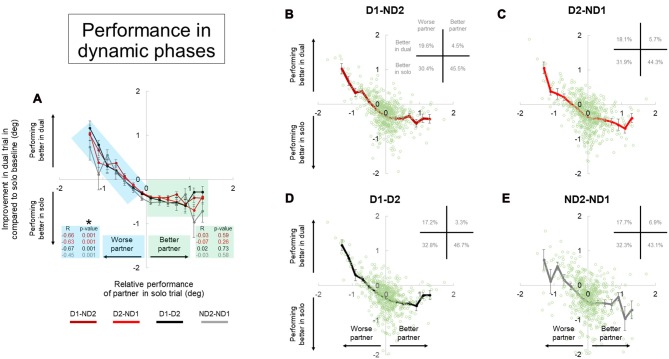
Performance during dual interaction vs. during bimanual interaction in dynamic phase. Influence of all dual interaction with respect to bimanual interaction is shown in **(A)**. The improvement in task performance in each subject for each dual trial was plotted against the relative performance of their partner. The dual trial improvement was measured by the change in tilt error by a subject during a single trial compared to his individual tilt error in the correspondent dual trial. The positive and negative abscissas in *x*-axis corresponds to a better (superior) performing partner and a worse (inferior) performing partner; respectively. The positive and negative abscissas in *y*-axis corresponds to an agent who is performing better in dual trial and performing better in solo trial; respectively. The green and blue shaded boxes are for better and worse partner; respectively. Average performances and all the data points are shown for each dual condition in **(B–E)**. Furthermore, the frequency of occurrence of data points in each quadrant is also shown in top right of each plot in **(B–E)**. Data are means averaged across all subjects. Vertical bars denote standard errors of the mean. The asterisk denotes statistically significant correlation or relationship (*p* < 0.05).

To quantify whether individuals manipulating an object with two hands (bimanual conditions) perform better than when interacting with another partner, we processed the performance data as follows: (1) we subtracted MAV(*e*) in the bimanual condition of subject 1 from MAV(*e*) in the bimanual condition of subject 2 to define the better bimanual performer, and assigned positive or negative values to the “better” and “worse” subject, respectively. For example, first and second partners have MAV(*e*) values of 0.3 and 0.7 in their bimanual condition. The performance difference between the two is ±0.4, so we assigned +0.4 to first partner (better partner) and –0.4 to second partner (worse partner). (2) For each participant, we subtracted MAV(*e*) of the dyadic condition from MAV(*e*) of his/her bimanual condition to define the extent to which a given subject performing the bimanual task improved his/her performance when partnering with another participant. Figures 8, 9 show plots of data obtained from steps (1) and (2) (*x-* and *y-*axis, respectively). To address the question of whether dyadic interaction may be beneficial or detrimental to the solo performance of each participant, we performed two separate linear regression analyses, one on the data from the “better partner” and the other on the data from the “worse partner” (see green and blue shaded data, respectively; Figures 8A, 9A).

## Results

### Learning Effect in Performance within Block for all Conditions

During the static phases of our manipulation task, analysis of object tilt revealed that individual agents (solos) performed the manipulation task better, i.e., generated less error, than dyads (main effect of *Condition*: *p* = 0.001). Specifically, the MAV of object tilt (MAV(*e*)) was significantly smaller for the Bi group than D1-D2 and ND2-ND1. We also found that participants improved their performance with practice, as performance error was significantly smaller in late than early trials (main effect of *Trial*: *p =* 0.001). There was no difference between bimanual groups (no main effect of *Group*: *p =* 0.902) and no significant interactions were observed in any combination of between and within-subject factors (all *p* > 0.05). The results of the analysis of object tilt during the dynamic phases were similar to those presented for the static phases (main effects of *Condition* and *Trial*: both *p* = 0.001; no *Group* effect: *p* = 0.953; no interactions for any factor combination: *p* > 0.05), except for pairwise comparisons revealing significantly smaller MAV(*e*)) for the Bi group than all the dyadic conditions (*p* < 0.05).

### Performance within Block Across Dyadic Conditions

The first statistical analysis using a repeated-measures ANOVA between two dyadic conditions consisting of both face-to-face configurations (D1-D2 vs. ND2-ND1) showed that there was no effect of Handedness in either dynamic (*Handedness*: *p* = 0.146; *Trial*: *p* = 0.007; Handedness*Trial: *p* = 0.936) or static phases (*Handedness*: *p* = 0.635; *Trial*: *p* = 0.006; Handedness*Trial: *p* = 0.862).

Mixed model analysis of the static phase revealed significant effects of *Configuration* (*p* = 0.002) and *Trial* (*p* = 0.001), but not *Handedness* (*p* = 0.985). Furthermore, pairwise comparisons showed that D1-D2 performance was significantly worse than both side-by-side conditions (D1-ND2: *p* = 0.015; D2-ND1: *p* = 0.037). Similarly, ND2-ND1 performance was significantly worse than side-by-side performances (D1-ND2: *p* = 0.018; D2-ND1: *p* = 0.049).

Mixed model analysis of the dynamic phase revealed significant effects of *Configuration* (*p* = 0.027) and *Trial* (*p* = 0.005), but not *Handedness* (*p* = 0.958). Furthermore, pairwise comparisons showed no significant difference between any pairwise comparison (all *p* > 0.05) with the exception of D1-D2 performance being significantly worse than D1-ND2 (*p* = 0.020).

To summarize, during static phases, subjects in both side-by-side participant configurations generated less error than both face-to-face configurations (main effect of *Configuration*; Figure 5A). Interestingly, this effect was not as consistent for dynamic phases, as better performance was found only for one face-to-face configuration relative to only one side-by-side configuration (Figure 5B). We should note that these significant effects of *Configuration* during both static and dynamic phases are nested with the handedness since the biomechanical grasp criterion (thumb inside the U-shape device) prevents us from making conclusive inferences about a “pure” effect of configuration. Most importantly, however, there was no effect of *Handedness* in face-to-face configurations when there was no confounding factor of nesting handedness and participant configuration in the analysis. Furthermore, supplementary analysis revealed that the superior performance of dyads in side-by-side configuration was associated with a smaller error (Figure 5A) and more zero line crossings in object orientation (Supplementary Figure S4A) than face-to-face configuration, and this was particularly evident in static but not dynamic phases (Supplementary Figure S4B).

### Performance Across Blocks for Each Experimental Condition (Practice Effect)

During static phases, participants from the bimanual, (Bi1, Bi2), D1-D2 and ND2-ND1 conditions (Figures 6A,C,D, respectively) did not exhibit learning across blocks of trials (all *p* > 0.05). However, D1-ND2 and D2-ND1 conditions were characterized by smaller performance error for later than earlier blocks (*R* = −0.13; *p* = 0.002). Therefore, the above-described main effect of practice on performance error underscores the sensitivity of the side-by-side configuration to practice. In contrast, during dynamic phases (Figure 7), there was no effect of practice in any experimental conditions even side by side condition (Figure 6B) did not show any significant trend. *P*-values of Bi, D1-ND2 and D2-ND1, D1-D2 and ND2-ND1 conditions were all >0.05. This indicates that the performance in dynamic phases of these condition does not differ if it is collected at the early or late blocks.

### Effect of Gender on Performance

We found no *Gender* effect or interactions with *Condition* or *Trial* in bimanual performance between male and female participants (dynamic phase: *p* = 0.113; static phase: *p* = 0.245). Furthermore, we found no difference in performance across dyads with mixed genders (*Gender combination*; dynamic phase: *p* = 0.089; static phase: *p* = 0.191; no interactions).

### Influence of Physical Interaction on Performance of Individual Agents

The improvement in task performance in each subject for each dyadic trial was plotted against the relative performance of their partner (Figures 8, 9). The performance improvement in the dyadic conditions relative to bimanual conditions was calculated as the difference in object tilt during dual and individual trials (see “Materials and Methods” section). In all dyadic conditions and regardless of task phase, on average performance of the “worse” partners improved linearly with respect to his/her baseline activity in the bimanual condition (blue shaded box, Figures 8, 9). For the “better” partner, the correlation between improvement in dual trial relative to solo baseline vs. relative performance of the partner in solo trials was not strong (green shaded box, Figure 8A). However; for the “worse” partner this correlation was significant for all the conditions (negative slope and *p* = 0.001; blue shaded box in Figure 8A).

Further examination of data distributions (plots **B–E**, Figures 8, 9) revealed that dyadic conditions elicited a better performance of the “worse” partner only in ~50.8% (±2.5) and 36.0% (±1.8) of the trials in static and dynamic phases, respectively. In contrast to the “worse” partners, performance of all “better” partners tended to deteriorate when performing the task with a partner (green shaded box, Figures 8, 9) in most trials (80.9 ± 2.9% and 89.7 ± 2.7% in static and dynamic phases, respectively; plots **B–E**, Figures 8, 9).

Examination of the percentages of trials in each plot quadrant (see inset in each plot of Figures 8, 9), the dynamic phase performance appears to be more detrimental to solos compared to static phase, such that: (1) the occurrence of being “better” partner and performing better in solos is increased (compare forth quadrants in Figures 8, 9); and (2) the occurrence of being worse partner and performing better in dyadic conditions is decreased (compare second quadrants in Figures 8, 9). In other words, when going from static to dynamic task phases, the percentages of trials in the first and second quadrants decrease by shifting to the third and fourth quadrants.

## Discussion

We examined the effects of interpersonal motor coordination of two agents through a physically-coupled object on performance of manipulation. Contrary to our hypotheses, handedness—tested by pairing of dominant hands and paring of non-dominant hands—did not have a significant influence on dyadic task performance. However, we did find effect of configuration when holding the object: subjects performed better when sitting side-by-side (D-ND and ND-D) than face-to-face (D-D and ND-ND). Most importantly, we demonstrated that the role of interpersonal coordination during physically-coupled joint actions is complex. Specifically, when two individuals are paired to manipulate an object, their joint performance is better than the bimanual performance of the worse partner, but worse than the bimanual performance of the better one. We also found that dyad configuration has an effect on manipulation performance. We discuss these results in the context of sensorimotor mechanisms and open questions for future research.

### Handedness Does Not Influence Motor Performance in Joint Actions

Handedness, as evaluated in terms of performance differences in dominant and non-dominant limbs, is thought to emerge from hemispheric lateralization (Serrien et al., 2006). It has been proposed that the left-hemisphere is specialized for controlling motor behaviors in familiar environments, i.e., predictive control, whereas the right-hemisphere is specialized for responding to unforeseen environmental events and signal dependent motor noise, i.e., impedance control (Sainburg, 2014). Specifically, predictive control is based on building accurate internal representations of the environment, which allows optimization of motor behavior (Haruno et al., 2001; Todorov, 2005) and produce consistent motion in a consistent environment. In contrast, impedance control could be accomplished by muscle co-activation (Burdet et al., 2001; Osu et al., 2009) and modulation of proprioceptive reflex gains (Mutha et al., 2008). Impedance of the arm/hand can be modulated to improve the end-point stability when errors in internal representations and motor noise arise during execution (Selen et al., 2009; Mitrovic et al., 2010). We hypothesized that hemispheric lateralization would influence the performance of joint actions of two individuals similarly. However, this hypothesis was not supported as we found no performance difference when comparing D-D and ND-ND conditions. We think that this result may point to important task differences that could account for the discrepancy between the limb dominance effects in previous studies and the present results. Specifically, typical reaching and unimanual object lifting tasks are often end-goal directed that involve rapid movements (typically ~500 ms). Additionally, these movements are usually performed in environmental conditions that may be mostly either predictable or unpredictable. Therefore, the hand that is specialized to perform best in one of these environmental conditions would have a performance advantage. However, both the dynamic and static phase of our task performed with two hands last much longer than previously used unimanual tasks (>2 s). This is because our task emphasized precision and required continuous monitoring of the behavioral outcome. Specifically, subjects were tracking a visual target (i.e., bubble level) throughout the entire trial to comply with the task requirement of keeping the base of the object horizontal. During this process, both agents cannot fully predict the consequence of the joint motor output as this is also a function of the other agent’s actions. Therefore, it is likely that our task might have engaged the interaction of predictive and impedance control mechanisms to a similar extent, thus overshadowing the hands’ role specialization. This interpretation is consistent with the finding by Kurillo et al. (2004) that handedness does not influence performance when subjects generated isometric finger force to track moving visual targets. However, it remains unclear how these two mechanisms interact throughout a trial as we only examined the average net motor outcome. One possibility is that they are engaged simultaneously to the same degree. That is, one can up-regulate impedance throughout the trial to compensate for the inaccurate prediction of the other agent’s actions, but not as much as the impedance used to stabilize the limb in response to completely unpredictable environment (Burdet et al., 2001). Alternatively, predictive and impedance control may occur intermittently. Such intermittency can be found in many tasks that requires continuous tracking of visual targets (Miall et al., 1993; Bye and Neilson, 2010). It has been argued that intermittency could arise from the visuomotor feedback loop delays (i.e., ~150 ms; Slifkin et al., 2000), or refractory period after motor corrections (Miall et al., 1993). In our tasks, subjects could choose to respond to an error either by predictive control or impedance control. However, our data cannot be used to conclusively distinguish between these two control mechanisms with high temporal resolution. Future studies have been planned to address this issue.

### Agents’ Configuration Influences Motor Performance during Static but Not Dynamic Task Phases

We found that the configuration of physically-interacting agents influenced motor performance in our manipulation task. Specifically, subjects performed better in side-by-side than in face-to-face configuration (Figure 5A). Additionally, side-by-side configuration exhibited a block order effect (Figure 6B), which suggests that performance in the later stage of the experiment benefited from having performed other task conditions, i.e., generalization. In contrast, no agents’ configuration effect was found in the dynamic phase.

We should note that, although the joint object manipulation task is similar across these two agents’ configurations in personal motor space, i.e., from the perspective of an individual agent, the visual space is drastically different. Specifically, side-by-side configuration closely resembles the bimanual configuration for both agents, as they see their own hand collaborating with a contralateral hand within each of their personal space. In contrast, face-to-face configuration involves visual image of another agent using the same hand. It has been proposed that the CNS of each individual predicts the action outcome of the partner through “simulation” with their own internal representations (Wolpert et al., 2003). Studies in magnetic stimulation, human and animal neuroimaging could support these ideas. For example, mirror neurons—a system for matching observation and execution of motor actions—are thought to engage in both self-generated actions and actions of others (Gallese et al., 1996; Rizzolatti and Arbib, 1998; Newman-Norlund et al., 2008; Ferrari et al., 2017). Furthermore, neural structures that are associated with action production also respond to imitation and observation of the same action generated by others (Grèzes et al., 2001; Fadiga et al., 2002). Interestingly, BOLD signal measured from areas in the human mirror system was stronger in joint-action conditions than when performing the task alone. Particularly, this activity is highly correlated with inter-dependence (level of complementary actions) of movements that cooperating individuals had to generate to fulfill a virtual balancing task. The demand on participants to simulate the actions of others might be reflected in the BOLD activity in mirror neuron system to generate appropriate responses by adapting their own actions with those of their partners (Newman-Norlund et al., 2008). This simulation or prediction process may involve the same feed-forward mechanisms supporting self-executed actions (Sacheli et al., 2012). Based on this consideration, we speculate that the observation of the partner using same hand may share the same neural resources used for controlling their own action, leading to interference in motor control during face-to-face coordination. This effect could be minimized during side-by-side coordination, as different hands are used. Interestingly, we found a weaker physical coupling in the face-to-face configuration, as this was characterized by a lower internal force than side-by-side configurations (see Supplementary Material and Supplementary Figures S1, S2). Such internal force has been interpreted as a potential communication channel between two interacting agents (Reed and Peshkin, 2008; van der Wel et al., 2011). It is possible that the aforementioned interference weakens dyad’s ability to enforce haptic channel in the face-to-face configuration. However, the exact link between spatial configuration and dyadic motor coordination patterns requires further investigation.

### Factors Affecting Performance Differences of Two Cooperating Agents vs. a Single Agent

Previous work on physical interactions has shown that dyads perform better than solo in some cases (Reed and Peshkin, 2008; Ganesh et al., 2014), or that they are performed equally well (van der Wel et al., 2011). As pointed out in the “Introduction” section, the number of end-effectors may play significant role in performance differences across solos and dyads. This is because the stiffness of each of the two end-effectors could add up to increase the stiffness of the whole (two-limb) system, thus reducing performance error. Therefore, a better performance in dyadic than unimanual action may not be entirely due to the fact that physical coupling or joint action adds an advantage to motor performance relative to manipulation performed by a single agent. By directly comparing inter- and intra- personal coordination, the result of our study demonstrated that performance of dyads is almost always worse than the performance of the better partner (Figures 8, 9, 4th quadrants), and is only sometimes better than the performance of the worse partner (Figures 8, 9, 2nd quadrants). This indicates that the ability of two brains to coordinate two end-effectors through physical coupling is mostly limited by the ability of the better partner. We discuss potential factors that could influence performance of coordination below.

#### Increased Uncertainty

Joint actions from two randomly-paired individuals could potentially induce environmental uncertainty. Specifically, when two brains are controlling effectors (one upper limb each), it is expected that either brain cannot fully predict the action of the other (Mojtahedi et al., 2017). Therefore, each individual may treat the motor output on the other handle as partially environmental noise or uncertainty. To assess how sensorimotor system estimate uncertainty, we used grip force as an indicator of increased uncertainty as it increases the safety margin (Hadjiosif and Smith, 2015). Indeed, subjects used higher grip force in dyadic conditions than bimanual conditions (see Supplementary Figure S3). This could explain why the dyadic performance was almost always worse than the better partner within our experiment. The effect of increased uncertainty may eventually reduce, but it would take many more trials to adapt to the partners for accurately predicting partner’s action. The elevated grip force reported here for interpersonal manipulation is also consistent with a recent study reporting larger grip forces for inter- than intrapersonal manipulation (Solnik et al., 2016).

#### Social Facilitation

Social facilitation, a factor that is specific to cooperative actions, has been defined as a tendency for individuals to perform differently when in the mere presence of others (Wegner and Zeaman, 1956; Schmitt et al., 1986; Sawers and Ting, 2014). Specifically, when individuals are aware of another agent being present during motor performance, they perform better than when others are not present (Wegner and Zeaman, 1956; Zajonc, 1965; Schmitt et al., 1986). For example, it has been reported that kinematics of reaching to grasp an object for placing it in an end target position is affected by whether the action is monitored or not by other agents (Fantoni et al., 2016). Social facilitation appears to play a role in performance differences between dyads and solos also when physical interactions are involved (Wegner and Zeaman, 1956), e.g., when subjects are aware that the agent they are cooperating with is a human agent (Wegner and Zeaman, 1956; Schmitt et al., 1986; Sawers and Ting, 2014). Thus, social facilitation might have played a role in enhancing the performance of the dyads relative to the worse partner in the current study. However, more work is needed to understand the physiological mechanisms elicited by social facilitation and the extent to which it contributes to better motor performance.

#### Sharing of Responsibility for Attainment of Common Motor Goals

Another reason why dyads in our study performed better than the worse partner is that two agents can share responsibility for attaining a common goal, while being in charge of controlling one effector instead of two as it happens in the bimanual task (Wegner and Zeaman, 1956; Knoblich and Jordan, 2003; Sawers and Ting, 2014). For example, dyads could perform better because each agent engages in one or more specific components of the task, e.g., one agent accelerates the crank during a movement phase while the other decelerates it on the subsequent phase (Reed and Peshkin, 2008). A similar interpretation was provided by another study on non-physical interaction tasks (Schmidt et al., 1998; Masumoto and Inui, 2013), suggesting that groups should be able to perform better than individuals since each person in a group can focus on a subset of the actions and have less individual responsibility during interactions. In our study, the worse partner may take a more “follower” type of role to focus on a subset of actions, therefore attain a greater degree of coordination. However, as pointed out above, the extent to which role asymmetry occurred in our task remains unclear and needs to be addressed by future experiments.

## Conclusion

The present work extends ongoing investigations aimed at evaluating performance during joint actions through physical coupling. Our findings reveal that agents’ configuration plays an important role in performance of joint actions, whereas handedness does not. Furthermore, we showed that the extent to which dyadic interactions may benefit performance is not a general rule as it is limited by the ability of the better partner. Ongoing neural imaging studies in our laboratory using the same experimental design is addressing mechanisms underlying physical joint interactions, which could potentially impact several areas, including robotic-assisted therapies, sensorimotor learning and human performance augmentation.

## Author Contributions

KM: data collection. KM, QF and MS: experimental design, data analysis and writing of manuscript.

## Conflict of Interest Statement

The authors declare that the research was conducted in the absence of any commercial or financial relationships that could be construed as a potential conflict of interest.

## Footnotes

^1^A similar presentation pattern was implemented for the other experimental condition, such as to have six subject pairs being tested on each condition across six blocks presented at different points during the experiment (Figure 4B).

## Supporting Information

We present the results of supplementary analysis of grip forces exerted during performance of the manipulation task as Supplementary Material in one PDF file. We also submitted all the data (object kinematic data from a motion tracking system; forces and torques data from 6-axis force/torques sensors) to the OSF (Open Science Framework) online repository or database in the following link: https://osf.io/3puex/?view_only=0a61d731af7b4a5fa4ef29bda31e9b02
