# Supplementary material for: On the Role of Physical Interaction on Performance of Object Manipulation by Dyads
Source: Front Hum Neurosci. 2017 Nov 7;11:533. doi: 10.3389/fnhum.2017.00533 (PMC5673979; doi:10.3389/fnhum.2017.00533)
Supplement: Supplementary file 1 [file Presentation_1.pdf]

Supplementary Material

On the role of dyadic interactions on performance of object manipulation

Keivan Mojtahedi, Qiushi Fu, Marco Santello*

*** Correspondence:** Dr. Marco Santello: Marco.Santello@asu.edu

We present here analysis of grip forces exerted during performance of the manipulation task. The first objective of our force analysis was to measure the internal force between the handles (**Supplementary Figure S1**). This analysis was motivated by addressing the question of whether spatial configuration, i.e. side-by-side versus face-to-face, of dyadic interactions might affect internal force between handles (**Supplementary Figure S2**), and grip force within each handle (**Supplementary Figure S3**). We consider internal force as a strategy to create a 'haptic channel' through which each agent can better infer the state of the cooperating agent's limb. In our framework, dyadic interactions are associated with higher uncertainty about the outcome of each agent's actions, as the resultant motion of the object is a function of actions of both agents, where the partner's actions are unpredictable.

Tangential forces (please refer to inset in **Figure 1**) were constrained to the object weight and countered the gravity force field. Hence grip or normal forces (please refer to inset in **Figure 1**; all grip forces also shown in **Supplementary Figure S1**) played key roles in establishing haptic channels or internal forces. **Supplementary Figure S1** showed the grip forces on the handles for index and thumb sides. Internal forces could be exerted inward or outward. For example, **Supplementary Figure S1** shows inward forces in which grip force of index side (GF_I) was larger than grip force of thumb side (GF_T) in each handle. So, in this example dyads are pushing against each other by generating inward forces.

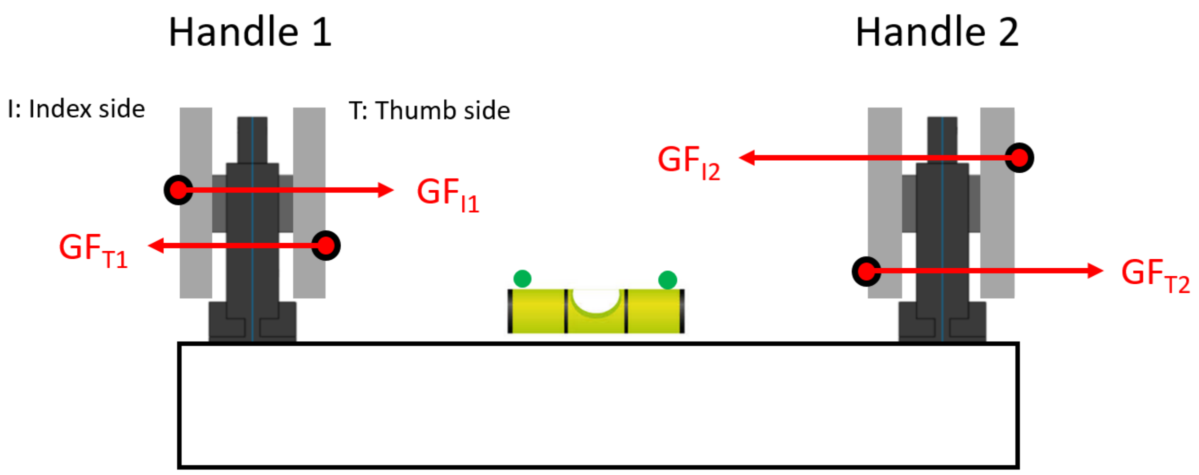

Supplementary Figure S1. Grip forces on the handles for index and thumb sides. Red dot on the grasping surface represents the center of pressure (*CoP*; red dot).

In **Supplementary Figure S2**, we show grip forces exerted on the index finger and thumb sides of Handle 1 and then calculated the absolute difference between the two (i.e. $|GF_{I1}\text{-}GF_{T1}|$). Similarly, we calculated this absolute difference in Handle 2 (i.e. $|GF_{I2}\text{-}GF_{T2}|$). As internal force is defined as the force components that has not net effect on the object (they cancel out), we computed the minimum of the above two absolute differences at each time sample to measure the amount of internal force between the handles ($Min\{|GF_{I1}\text{-}GF_{T1}|\ ,\ |GF_{I2}\text{-}GF_{T2}|\}$). Lastly, we calculated the average of the internal force across times for static or dynamic phases (**Supplementary Figure S2(A,B)**, respectively).

We also performed another ANOVA with repeated measures on $Min\{|GF_{I1}\text{-}GF_{T1}|\ ,\ |GF_{I2}\text{-}GF_{T2}|\}$ for dyadic conditions using two within-subject factors: *Trial* (2 levels: trials 1-4 and trials 5-8), and *Configuration* (2 levels: side-by-side (D1-ND2 and D2-ND1) and face-to-face (D1-D2 and ND2-ND1)). Statistical results revealed that internal force in side-by-side configuration was significantly greater than face-to-face configuration in both static and dynamic phases (no effect of *Trial*: $p > 0.05$, main effect of *Configuration*: $p = 0.001$).

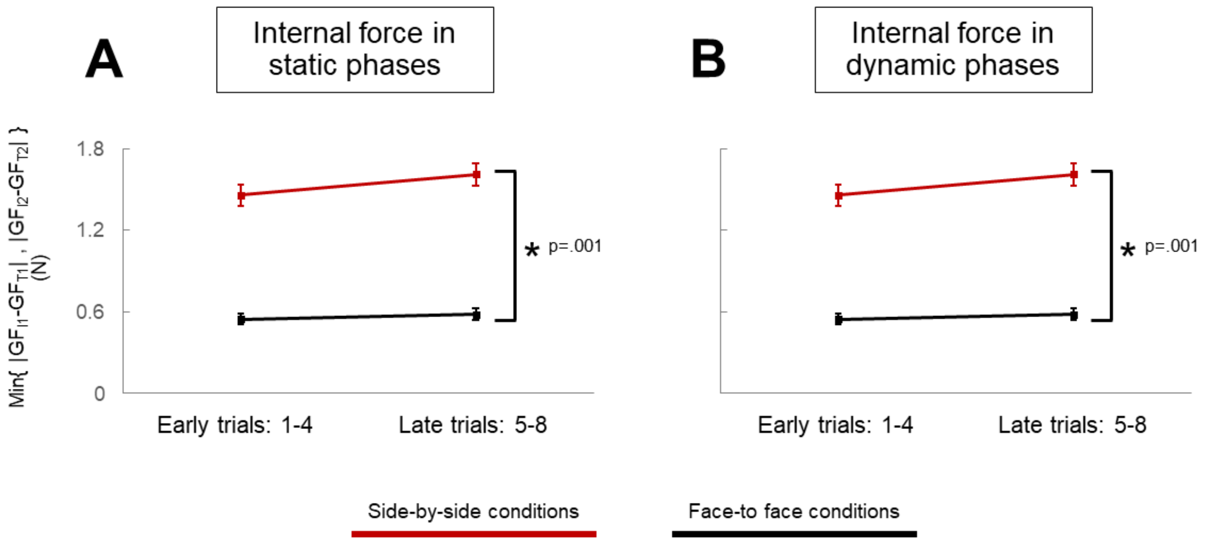

Supplementary Figure S2. Internal force in dyadic interactions: side-by-side and face-to-face configurations.

We consider grip force as a measure of subjects' response to uncertainty. **Supplementary Figure S3** shows grip force of each handle averaged across time samples in static or dynamic phases. We denoted the mean value of (GF_I+GF_T) over time for handle 1 as $\mathrm{GF_{H1}}$. Then, we averaged these two handle average values, i.e. $\mathrm{GF_{Avg}} = (\mathrm{GF_{H1}} + \mathrm{GF_{H2}})/2$. The average GF ($\mathrm{GF_{AVG}}$) represents the average amount of grip force which was generated on the handles or the average grip force level during interaction (**Supplementary Figure S3**).

To assess learning within each block of trials (i.e., experimental condition), we divided the 8 trials into "Early trials" (trials 1-4" and "Late trials" (trials 5-8). We performed analysis of variance (ANOVA) with repeated measures on average GF ($\mathrm{GF_{Avg}}$) using one between-subject factor, *Group* (2 levels: Bi1 and Bi2), and two within-subject factors: *Trial* (2 levels: trials 1-4 and trials 5-8), and *Condition* (5 levels: Bi, D1-ND2, D2-ND1, D1-D2, ND2-ND1).

During the static phases of our manipulation task, analysis of grip force revealed that individual agents (solos) generated less grip force than all dyads (main effect of *Condition*: p = 0.001). We also found that participants decreased their forces with practice, as it was significant smaller in late than

early trials (main effect of *Trial*: $p = 0.035$). There was no difference between bimanual groups (no main effect of *Group*: $p = 0.954$) and no significant interactions were observed in any combination of between and within-subject factors (all $p > 0.05$). All pairwise comparisons revealed significantly smaller grip force for the Bi group than all dyadic conditions (all $p < 0.05$) and no difference among dyadic conditions (all $p > 0.05$).

The results of the analysis of GF_{Avg} during the dynamic phases were similar to those presented for the static phases (main effects of *Condition*: $p = 0.001$ and *Trial*: $p = 0.044$; no *Group* effect: $p = 0.941$; no interactions for any factor combination: $p > 0.05$). All pairwise comparisons revealed significantly smaller GF_{Avg} for the Bi group than all the dyadic conditions ($p < 0.05$) and no difference among dyadic conditions (all $p > 0.05$).

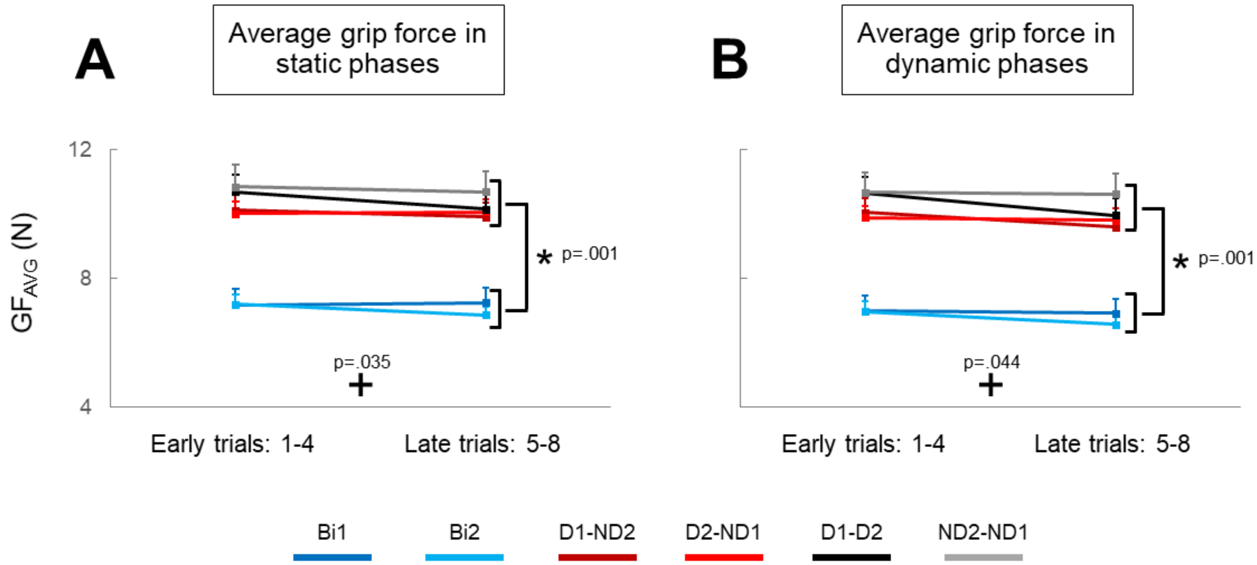

Supplementary Figure S3. Average grip force level across all conditions.

We also provided the results for time-normalized zero crossings of error per second (NZCE/s) to assess the sensitivity of object orientation control relative to the desired (horizontal) orientation across all static and dynamic phases of each trial.

We performed repeated measured ANOVA on dyadic conditions using two within-subject factors (*Trial* and *Condition*). We found no significant effect in dynamic phase. However, we found a significant effect of both *Trial* and *Condition* in the static phase. The time course of error in the side-by-side configurations was characterized by more zero line crossings than face-to-face configurations. The superior performance of dyads in side-by-side configurations was associated with a smaller error (**Figure 5A**) and more zero line crossing in object orientation (**Supplementary Figure S4A**) than face-to-face configuration particularly in static phase, but not dynamic phase (**Supplementary Figure S4B**).

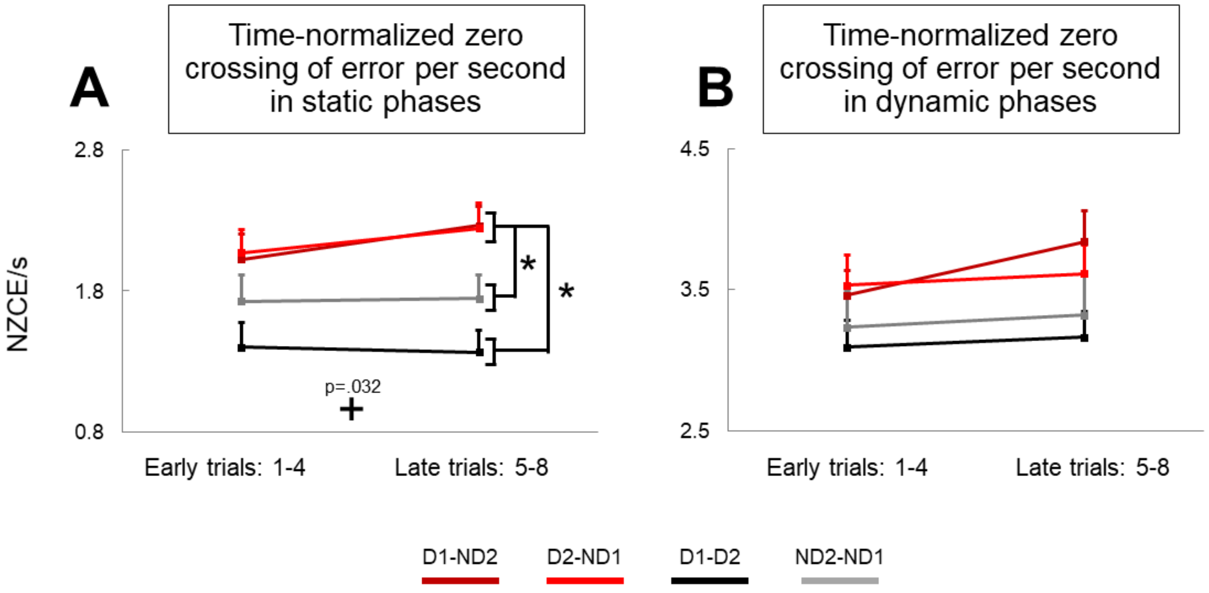

Supplementary Figure S4. Time-normalized zero crossing of error (e) per second (NZCE/s) was measured on early and late trials for both dynamic and static phases. Data are means averaged across all subjects. Vertical bars denote standard errors of the mean. The symbol “+” indicates a statistically significant Trial effect. The asterisk denotes statistically significant differences ($p < 0.05$).
